# Linking EMT Dynamics to Cellular Plasticity: YAP/TAZ as Central Regulators Across Physiological and Pathological States

**DOI:** 10.3390/genes17080851

**Published:** 2026-07-24

**Authors:** Laura Amicone, Carla Cicchini, Fabio Petti, Alessandra Marchetti

**Affiliations:** Department of Molecular Medicine, Sapienza University of Rome, Viale Regina Elena 324, 00161 Rome, Italy; laura.amicone@uniroma1.it (L.A.); carla.cicchini@uniroma1.it (C.C.); f.petti@uniroma1.it (F.P.)

**Keywords:** hybrid epithelial/mesenchymal states, mechanotransduction, stemness, transdifferentiation, tissue repair, fibrosis, cancer

## Abstract

Cellular plasticity allows organisms to adapt dynamically to both physiological and pathological contexts. Epithelial–mesenchymal transition (EMT) is a well-known example of this plasticity and is now widely recognized as a reversible and highly dynamic spectrum of cellular states rather than a simple binary switch. In this review, we summarize current knowledge on the Hippo pathway transcriptional co-activators YAP and TAZ, focusing on their role as a central hub that integrates mechanical, biochemical and metabolic signals from the microenvironment to control cell fate reprogramming. We discuss how YAP/TAZ interact with EMT-related signaling pathways and transcriptional networks to regulate the acquisition, maintenance and dynamic remodeling of mesenchymal states, as well as hybrid epithelial/mesenchymal (E/M) phenotypes. We also highlight the presence of interconnected feed-forward and feedback regulatory loops within the YAP/TAZ–EMT axis, which contribute to the stabilization of cellular plasticity and support context-dependent transcriptional programs. These mechanisms are involved in key physiological processes, including embryonic development and tissue repair, and in pathological conditions such as organ fibrosis and cancer progression. Therefore, we propose a model where YAP/TAZ act as the central molecular hub within the networks governing cellular plasticity and EMT dynamics. Finally, we discuss how a better understanding of the mechanistic basis of YAP/TAZ-driven EMT may provide a useful framework for the development of therapeutic strategies aimed at modulating cellular plasticity in cancer, fibrotic diseases and regenerative medicine.

## 1. Introduction

Cellular plasticity is a fundamental feature that enables cells to dynamically reprogram their identity, function and behavior in response to both genetic programs and microenvironmental signals. During embryonic development, this plasticity is essential for the coordinated lineage specification and morphogenetic processes that drive the formation of complex tissues and organs [1]. In adult organisms, it contributes to tissue homeostasis and regeneration by allowing resident stem/progenitor cells and, in some conditions and contexts, differentiated cells to respond to physiological demands and contribute to tissue repair [2].

Cellular plasticity is also involved in disease onset and progression. In fibrosis and chronic inflammatory disorders, indeed, persistent microenvironmental cues can lead to sustained cell-state transitions that result in tissue remodeling, persistent inflammation and progressive organ dysfunction [3]. In cancer, cellular plasticity drives the transition of tumor cells between different phenotypic states which enable them to invade, metastasize, develop therapy resistance or evade immune surveillance as well as promote tumor recurrence [4]. Therefore, while cellular plasticity appears crucial for embryonic development and tissue repair, its imbalanced regulation can lead to the acquisition of aberrant cell phenotypes with a key role in pathological states.

In both physiology and pathology, cellular plasticity represents a dynamic and tightly regulated trait of many cell types, orchestrated by complex signaling networks integrating mechanical, biochemical, and metabolic inputs. Among the pathways governing this adaptive response, the Hippo signaling pathway is established as a central regulator, with Yes-associated protein (YAP) and its paralog Transcriptional co-Activator with PDZ-binding motif (TAZ) acting as its major transcriptional effectors. The core of the mammalian Hippo pathway contains a kinase signaling cascade, formed by the serine/threonine kinases MST1/2 and LATS1/2. When the Hippo pathway is turned on, MST1/2 phosphorylates and activates LATS1/2, which in turn phosphorylates YAP and TAZ. This phosphorylation promotes their cytoplasmic sequestration and ubiquitin-mediated, proteasome-dependent degradation, thereby preventing their translocation into the nucleus and the interaction with the main transcriptional partner TEAD to regulate gene expression [5]. Therefore, the canonical Hippo signaling promotes the inactivation of YAP/TAZ, limiting transcriptional programs involved in proliferation, survival, stemness and cellular reprogramming [6]. However, a wide range of mechanical, biochemical and metabolic cues can activate YAP/TAZ through both Hippo pathway inhibition and Hippo-independent mechanisms. As a result, YAP and TAZ integrate diverse environmental inputs and translate them into transcriptional programs that control cell reprogramming and adaptive changes in cell identity [7].

One of the best-characterized examples of cellular plasticity is the EMT, a biological process in which epithelial cells progressively lose their polarity and cell–cell adhesions while acquiring mesenchymal traits and enhanced motility [8,9]. Although EMT was initially described as a binary transdifferentiation from an epithelial to a mesenchymal state, it is now recognized as a dynamic transition across a continuum of phenotypes, highlighting its complexity [10]. Cells undergoing EMT frequently adopt intermediate or hybrid E/M phenotypes characterized by the simultaneous expression of both epithelial and mesenchymal markers. These partial E/M states reflect a fine-tuned and dynamic process, in which cells do not necessarily complete a full phenotypic conversion; instead, they can occupy stable or metastable intermediate configurations and, in some cases, acquire distinct functional properties, including enhanced adaptability to microenvironmental changes [11,12,13].

An increasing body of evidence has highlighted the key role of YAP/TAZ in the regulation of EMT and its intermediate cell states, positioning them at the center of the regulatory network governing epithelial–mesenchymal dynamics in both physiological and pathological conditions. Here, we review this expanding role of YAP/TAZ as a central hub that integrates mechanical and biochemical cues to orchestrate cellular plasticity. We examine the bidirectional circuitry linking YAP/TAZ activity and EMT and its contribution to morphogenesis, tissue remodeling, regeneration, fibrosis and cancer. Furthermore, we discuss how the knowledge about the mechanisms involved in the YAP/TAZ-driven EMT and cell plasticity can impact on therapeutic approaches.

## 2. YAP/TAZ as Regulators of Cellular Plasticity

YAP and TAZ have emerged as central regulators of cellular plasticity by integrating mechanical, spatial, metabolic and biochemical signals. These inputs shape cell behavior across embryonic development, adult tissue homeostasis, regeneration, and disease. As mechanosensitive transcriptional co-activators, YAP/TAZ respond to extracellular matrix (ECM) stiffness, cell geometry, cytoskeletal tension, cell–cell interactions, growth factor signaling, and metabolic cues through dynamic nucleo-cytoplasmic shuttling; once in the nucleus, they primarily associate with TEAD transcription factors to induce transcriptional programs that govern cell fate decisions, progenitor competence, tissue growth, and adaptive responses [7,14].

During embryonic development, YAP converts positional and mechanical information into lineage-specific transcriptional programs. In the pre-implantation embryo, Hippo signaling spatially restricts YAP activity: nuclear YAP accumulates in outer cells to promote trophectoderm specification via TEAD4 and the activation of trophectoderm genes (for example, Cdx2), whereas nuclear exclusion in inner cells preserves inner cell mass identity [15,16]. Beyond early lineage segregation, YAP/TAZ maintain progenitor plasticity in multiple developing tissues by sustaining proliferative competence and coordinating fate decisions in response to morphogenetic cues, by coupling microenvironmental inputs to transcriptional regulation, throughout organogenesis [7,17].

In adult tissues, YAP/TAZ activation not only regulates proliferation but also orchestrates adaptive changes in cell identity that enable differentiated cells to transiently re-acquire progenitor-like features during tissue repair. In the liver and other epithelial contexts, YAP/TAZ promote reversible dedifferentiation programs, expansion of facultative progenitor populations and tissue repair following injury [18,19]. These regenerative effects are often independent of a full EMT; rather than enforcing a terminal mesenchymal fate, YAP/TAZ establish a permissive transcriptional and epigenetic state that enhances cellular competence and responsiveness to environmental cues [19]. Notably, whereas transient YAP/TAZ activation restores homeostasis, sustained activation blocks cells into persistent progenitor-like states, driving uncontrolled proliferation, and hyperplastic or tumorigenic phenotypes [17,18].

Beyond established roles in stemness and regeneration, growing evidence indicates that YAP/TAZ can actively drive lineage reprogramming and transdifferentiation in fully differentiated mammal cells [20]. Mechanistically, YAP/TAZ cooperate with chromatin-remodeling complexes such as SWI/SNF, to reshape the epigenetic landscape. Making previously repressed regions accessible, YAP/TAZ reduce lineage restrictions and lower energetic barriers to fate stability, rendering cells highly sensitive to local morphogenetic cues (e.g., Wnt or BMP gradients) that can redirect them toward alternative lineages [21,22]. Consistently, co-expression of YAP with pluripotency factors, such as OCT4 and SOX2, facilitates the acquisition of pluripotent or progenitor-like states, highlighting its role in modulating cellular competence [23].

Together, these findings identify YAP/TAZ as master regulators of cellular plasticity, integrating microenvironmental signals with transcriptional and epigenetic programs to modulate cell identity, regenerative potential and phenotypic flexibility across development, tissue homeostasis and disease.

## 3. EMT Plasticity and Hybrid E/M States

EMT is a highly dynamic and reversible transdifferentiation program through which epithelial cells progressively lose their characteristics to acquire mesenchymal features, acquiring migratory capacity, invasiveness and environmental adaptability [8]. While EMT is essential for development and tissue repair, its aberrant activation contributes to pathological conditions such as fibrosis and cancer progression [9].

Although EMT was originally described as a binary process in which epithelial cells transdifferentiate into mesenchymal cells, accumulating body of evidence has revised this view. EMT is now recognized as a dynamic and reversible continuum of phenotypic states, rather than a direct conversion of one differentiated cell type into another differentiated one, where transitional cells encompass a range of intermediate configurations known as partial EMT (pEMT) or hybrid E/M states. These intermediate phenotypes are characterized by the simultaneous expression of epithelial traits, such as cell–cell adhesions and apico-basal polarity, and mesenchymal features including motility, cytoskeletal remodeling and invasive potential [10,24,25].

The existence of hybrid E/M states reflects the capacity of cells to finely tune their identity in response to microenvironmental cues, including ECM composition, mechanical stress, hypoxia, inflammatory signals, and paracrine factors [26]. Rather than transient intermediates, these hybrid phenotypes can form stable or metastable cellular states with unique functional properties. Hybrid E/M cells are critical drivers of collective migration, maintaining intercellular adhesion while acquiring migratory capabilities for coordinated cell dissemination [11]. In cancer, pEMT states have been associated with enhanced metastatic potential, stem cell-like characteristics, immune evasion and multidrug resistance [27,28,29]. In organ fibrosis, E/M cells do not show an invasive phenotype, while they acquire the ability to synthesize ECM components [30].

From a systems biology perspective, EMT can be viewed as a nonlinear regulatory network in which signaling pathways, master transcription factors (EMT-TFs), epigenetic regulators and post-translational mechanisms interact to generate and maintain phenotypic identities [31,32].

EMT-TFs, such as SNAIL (SNAI1), SLUG (SNAI2), Zinc-finger E-box-binding homeobox (ZEB) factors 1 and 2 (ZEB1 and ZEB2), TWIST family of basic helix–loop–helix factors (TWIST1 and TWIST2) and Paired-related homeobox 1 (PRRX1), interact within nonlinear regulatory circuits to generate multiple stable and metastable states [11,32,33]. The transcriptional activity of EMT-TFs is integrated with extensive epigenetic modifications [34,35] and post-transcriptional control by noncoding RNAs, such as the miR-200 family members [36,37] and long-noncoding RNAs like HOTAIR [38,39] and MALAT1 [40,41].

Within this framework, partial EMT emerges from the balanced activation of epithelial and mesenchymal gene programs rather than the complete dominance of either phenotype. Recent evidence indicates that the activity of distinct EMT-TFs as well as the balance between them can determine the functional properties acquired by transitional cells. Interestingly, Youssef and Colleagues [42] showed that SNAIL functions as an early regulator associated with inflammatory and plasticity-related programs, whereas PRRX1 favors the acquisition of more stable invasive mesenchymal traits. Notably, both paths remain plastic and interdependent, allowing dynamic transitions between different phenotypic states [42]. This phenotypic balance is regulated by hierarchical transcriptional circuits integrating extracellular signals with EMT programs, as exemplified by the regulatory circuit between Transforming growth factor beta (TGFβ), SNAIL and PRRX1. Transient TGFβ exposure induces SNAIL activation and favors hybrid E/M states, whereas prolonged stimulation promotes PRRX1 activation and reinforces mesenchymal commitment through additional regulatory mechanisms, including repression of SNAIL [43]. Thus, the duration and intensity of EMT-inducing signals influence whether cells remain in a plastic hybrid E/M state or progress toward a more committed mesenchymal phenotype.

The EMT plasticity is further sustained by epigenetic mechanisms that maintain EMT regulators in a poised and reversible state. Stable intermediate EMT states have been associated with bivalent chromatin configurations at the promoters of key EMT transcription factors, including SNAIL and ZEB1, which contributes to the maintenance of epithelial–mesenchymal plasticity and preserves cell responsiveness to persistent environmental cues [44,45]. In addition, chromatin-modifying complexes were shown to critically influence EMT trajectories. While both PRC2 and KMT2D-COMPASS are required to maintain epithelial cell identity, their selective dysfunction directs cells toward distinct EMT programs, with PRC2 loss promoting a high metastatic mesenchymal state [46].

Emerging evidence suggests that the maintenance of EMT plasticity results in the establishment of negative regulatory circuits counterbalancing reinforcing positive feedback [47]. Mutually inhibitory networks such as miR-200/ZEB and miR-34/SNAIL, together with phenotypic stability factors including GRHL2, OVOL1/2, and NRF2, are thought to limit irreversible lineage commitment while buffering stochastic fluctuations [48,49,50,51,52].

Together, these findings highlight that EMT-associated plasticity is governed by coordinated transcriptional, epigenetic and post-transcriptional regulatory networks, in which positive and negative feedback mechanisms cooperate to stabilize hybrid E/M states while preserving the phenotypic flexibility required for adaptation to changing environmental conditions. However, evidence demonstrating that these mechanisms suppress transcriptional noise and maintain metastable phenotypic states in vivo remains limited.

## 4. YAP/TAZ-EMT: A Bidirectional Regulatory Circuit Shaping Cell Fate

Accumulating evidence indicates that EMT- and YAP-induced transcriptional programs are interconnected through a complex bidirectional regulatory network. EMT and YAP activity, indeed, mutually influence each other, generating feedback mechanisms that contribute to the acquisition and maintenance of cellular plasticity. Through these reciprocal interactions, EMT-related regulatory pathways can promote and maintain YAP/TAZ activation, while YAP/TAZ-driven transcriptional programs can, in turn, enhance EMT-associated cellular processes. This positive feedback loop reinforces mesenchymal characteristics and helps stabilize intermediate epithelial–mesenchymal states. Understanding this dynamic crosstalk is therefore essential to elucidate how cells establish and maintain partial or fully mesenchymal phenotypes in both physiological and pathological contexts.

### 4.1. YAP/TAZ Activation in EMT

The EMT process is triggered by a wide range of biochemical and biomechanical stimuli from the microenvironment, which collectively orchestrate the activation of mesenchymal transcriptional programs and the repression of the epithelial one [53]. These multifaceted inputs frequently converge on YAP/TAZ activation, facilitating nuclear translocation and transcriptional outputs, often through the suppression of canonical Hippo signaling.

#### 4.1.1. Mechanical Cues

Biomechanical forces are fundamental determinants of epithelial–mesenchymal dynamics. Variations in the physical properties of the extracellular niche trigger and sustain EMT, while the transition itself actively reshapes tissue mechanics through reciprocal ECM remodeling [54]. The actin cytoskeleton acts as the primary transducer of these mechanical inputs, coupling physical tension to phenotypic reprogramming [55].

Mechanical perturbations represent some of the most potent activators of YAP/TAZ [56]. Elevated ECM rigidity, cell spreading, cyclic stretching, and actomyosin-mediated tension collectively drive the nuclear accumulation of YAP/TAZ and transcriptional activity [57,58,59].

Integrin engagement with a stiffened ECM activates Focal Adhesion Kinase (FAK), Src kinases, and RhoA/ROCK signaling, promoting actin polymerization and intracellular tension required for YAP/TAZ nuclear translocation [57,60,61]. Additionally, membrane sensors like Caveolin-1 (CAV1) can modulate YAP responsiveness to ECM stiffness by controlling actin dynamics and Rho-dependent tension, independently of the Hippo pathway [62].

Beyond static mechanics, dynamic physical forces significantly influence the YAP-dependent transcriptional programs. In mesothelial contexts, cyclic mechanical stretch promotes YAP/TAZ activation, driving mesothelial-to-mesenchymal transition characterized by upregulation of mesenchymal markers and ECM remodeling enzymes [63]. Similarly, fluid shear stress engages YAP signaling to enhance migratory capacity [64] and EMT [65], thereby promoting the invasive potential of cancer cells.

This mechanotransduction axis is also a pivotal regulator of hepatic cell fate. A soft ECM, mimicking the physiological/healthy liver state, restricts YAP activity promoting progenitor differentiation and supporting epithelial stability. Conversely, a rigid ECM, reflecting fibrotic pathology, triggers YAP activation and maintains progenitor-like and mesenchymal traits [66]. Notably, YAP directly modulates liver cell identity by inducing the EMT transcription factor SNAIL while suppressing the hepatocyte master regulator HNF4α [67], demonstrating that YAP/TAZ translate mechanical information into adaptive transcriptional programs that regulate cell fate commitment and differentiation.

#### 4.1.2. Biochemical Cues

YAP/TAZ activation during the EMT continuum is further amplified by its extensive crosstalk with major signaling pathways governing cell identity.

TGFβ is established as the primary driver of EMT, activating via SMAD-dependent and non-canonical signaling (including MAPK, PI3K, and Rho GTPases) to induce master EMT-TFs and downregulate epithelial markers like E-cadherin [68,69].

Beyond the direct transcriptional regulation, TGFβ signaling actively promotes YAP/TAZ activation. In the canonical cascade, activated SMAD complexes physically associate with YAP/TAZ in the nucleus [70] to cooperatively regulate genes involved in stemness, migration and cellular plasticity [71,72,73]. Concurrently, TGFβ stimulates non-canonical pathways, such as the RhoA-mediated cytoskeletal remodeling, promoting YAP/TAZ nuclear localization via Hippo-independent mechanisms [74,75]. Additionally, TGFβ signaling through the MEK5–ERK5 axis has been shown to sustain YAP-dependent transcriptional programs [76,77].

In addition to TGFβ, the Wnt/β-catenin and growth factor pathways converge on YAP/TAZ. In the absence of Wnt ligands, YAP/TAZ are sequestered within the β-catenin destruction complex (APC/Axin/GSK3β/β-TrCP) and targeted for proteasomal degradation. Upon Wnt stimulation, the disruption of this complex facilitates the stabilization and accumulation of both β-catenin and YAP/TAZ into the nucleus, where cooperatively they amplify proliferation and stemness gene programs [78,79,80] and coordinate tissue regeneration after injury [81]. Notably, β-catenin can directly induce YAP expression in colorectal cancer [82], suggesting the presence of a positive feedback loop that sustains the transcriptional output of both protein pathways. Conversely, active Hippo signaling can antagonize Wnt activity by promoting YAP/TAZ binding to β-catenin in the cytoplasm, thus preventing the nuclear translocation [83].

Receptor tyrosine kinases (RTKs), activated by growth factors such as HGF, FGF, and EGF, can trigger partial or complete EMT by upregulating core EMT-TFs [84]. These growth factors also promote YAP/TAZ activation via MAPK/ERK- or PI3K/AKT-mediated inhibition of the Hippo cascade, as well as through Src-dependent tyrosine phosphorylation which enhances YAP nuclear occupancy and transcriptional function [85,86,87].

Microenvironmental stressors such as hypoxia and inflammation further reinforce YAP/TAZ activation and EMT induction.

Hypoxia has emerged as a critical driver of EMT through the integration of multiple transcriptional and signaling circuits [88], including both Hippo-dependent and -independent mechanisms of YAP/TAZ activation. Hypoxia modulates Hippo signaling by promoting the degradation of key pathway components, leading to YAP/TAZ activation [89]. Moreover, under low oxygen conditions, stabilized HIF-1α physically interacts with YAP to favor its nuclear localization and promote programs associated with survival, invasion, and metabolic adaptation [89,90]. YAP also limits DNA damage and apoptosis during hypoxic stress [91] and regulates metabolism by promoting glycolysis and stem-like properties [85]. Collectively, the hypoxia-HIF-1α-YAP/TAZ axis establishes a feed-forward circuit that amplifies adaptive plasticity under low oxygen conditions.

Inflammation favors EMT through cytokines such as TNF-α, IL-6, and IL-1β [92]. Inflammatory cues frequently induce YAP activation either directly via cytokine-dependent pathways [93] or indirectly through inflammation-driven cytoskeletal tension and matrix stiffening [94]. Specifically, IL-6 family cytokines can activate YAP through gp130-dependent Src signaling, linking inflammatory responses to tissue repair [95]. Moreover, YAP/TAZ contribute to the activation of the JAK-STAT3 axis in cancer, enhancing inflammatory signaling and tumor progression [96]. Notably, YAP/TAZ functionally cooperates with STAT3 to regulate the transcription of genes involved in cell survival, migration and neoplastic transformation [97].

Finally, Notch-Delta signaling contributes to EMT [98] and sustains YAP activity upregulating its expression [99]. In turn, YAP/TAZ enhance Notch signaling by inducing receptors (e.g., Notch2) and ligands [19,100], thus promoting pathway activation either cell-autonomously or in neighboring cells. Moreover, nuclear YAP/TAZ and Notch-dependent transcriptional complexes cooperatively regulate common targets involved in progenitor maintenance, proliferation and lineage commitment both during early embryogenesis and in adult cell contexts [101,102].

### 4.2. YAP as a Driver and Stabilizer of EMT Through Positive Feedback Loops

YAP is not only a mediator and sensor of EMT-inducing signals from the microenvironment, but also a key driver and stabilizer of EMT program, coordinating the activation of the mesenchymal transcription and by contributing to the maintenance of cell state transitions via positive feedback loops.

Although YAP and TAZ proteins lack direct DNA-binding domains, they interact physically and cooperate with EMT-TFs to induce mesenchymal programs. For instance, YAP functionally cooperates with ZEB1, converting it from a transcriptional repressor to a transcriptional activator [103]. YAP/TEAD/ZEB1 complex promotes the expression of genes associated with stemness, invasion and aggressive tumor behavior, thereby reinforcing EMT and cellular plasticity [103,104,105]. Interestingly, ZEB1 reinforces this axis by the transcriptional repression of WWC1/KIBRA, an essential scaffold protein that promotes LATS kinase activation [106]. Loss of WWC1/KIBRA attenuates Hippo pathway activity and contributes to sustained YAP activation during EMT [107].

Other studies have demonstrated that YAP can actively promote EMT-TF expression. Noce et al. [67] showed that YAP directly contributes to SNAIL transcription in liver progenitor cells, establishing a positive regulatory loop that sustains mesenchymal commitment by reinforcing the downregulation of epithelial junctional components. Moreover, YAP was shown to directly transcriptionally activate SLUG expression with the subsequent E-cadherin downregulation in promoting EMT and migration/invasion of colorectal cancer cells [108]. Notably, YAP-mediated repression of epithelial differentiation programs can involve the inhibition of tissue-specific master transcription factors, such as HNF4α in liver cells. YAP can suppress HNF4α expression both directly and indirectly through SNAIL-dependent mechanisms, thereby promoting dedifferentiation and the acquisition of mesenchymal traits [67]. In turn, the loss of HNF4α removes its repressive transcriptional control over both YAP and SNAIL [67,109], establishing a positive feedback loop that further reinforces EMT progression [110]. Notably, microenvironmental cues, such as TGFβ, can further impair the activity of HNF4α and other members of the hepatocyte differentiation network [111,112], thereby shifting the regulatory balance toward sustained EMT activation.

In addition to the regulation of canonical EMT transcription factors, YAP also exerts part of its pro-EMT activity through other downstream transcriptional effectors. Among these, WT1 has emerged as an important mediator of EMT-associated transcriptional reprogramming. WT1 expression is induced by YAP and contributes to the repression of epithelial features, including E-cadherin expression, while promoting mesenchymal traits and migratory behavior [113]. Although in mesothelial cell systems the WT1/miR-769-5p axis has been shown to promote the reversion of mesenchymal-like states and restore differentiated characteristics [114], in most YAP-driven EMT models WT1 predominantly functions as a pro-mesenchymal effector [115]. Moreover, WT1 can be induced by EMT-promoting signaling pathways, particularly TGFβ signaling [116], placing it at the convergence of multiple EMT regulatory networks. Through the regulation of cell–cell adhesion, the YAP-WT1 axis contributes to the generation of a positive feedback loop in which the reduced epithelial cohesion (i.e., E-cadherin downregulation) further promotes YAP activation [117].

YAP can also induce the expression of AXL [118], a key receptor tyrosine kinase regulating cell invasion and supporting EMT and epithelial plasticity [119,120]. Of note, AXL can promote YAP activation through an EGFR-mediated LATS inhibition [121], thus establishing a regulatory loop reinforcing the EMT transcriptional program.

Finally, YAP/TAZ-mediated remodeling of the physical microenvironment creates a self-sustaining mechano-transductive circuit. Through TEAD-dependent transcriptional programs, YAP/TAZ induce the expression of genes involved in actin dynamics, focal adhesion turnover and ECM remodeling, including the matricellular proteins CTGF and CYR61 [57,58]. By driving matrix deposition and cytoskeleton reorganization, YAP/TAZ actively reinforce the same physical traits required to trigger its initial nuclear translocation [122].

Collectively, these findings indicate that YAP/TAZ are not merely downstream effectors of EMT but rather central transcriptional co-regulators that drive, reinforce and stabilize EMT programs through multiple feed-forward and feedback mechanisms, contributing to the persistence of partial and full mesenchymal phenotypes associated with increased cellular plasticity and adaptability.

## 5. Context-Dependent Roles of YAP/TAZ in EMT

### 5.1. YAP/TAZ in Developmental EMT (Type 1 EMT)

EMT plays a fundamental role during embryogenesis, particularly for mesoderm formation and neural crest delamination, where its molecular and cellular mechanisms have been extensively characterized. Developmental EMT is a dynamic, tightly controlled and reversible process driving organogenesis through extensive tissue remodeling and cell migration. In this context, YAP/TAZ act as key regulators of cellular plasticity, integrating mechanical cues (tension, matrix stiffness, tissue architecture) with the transcriptional programs of primary EMT.

Developmental EMT is highly coordinated in space and time, and YAP/TAZ activity reflects this precision by coupling morphogenetic forces to gene expression programs to shape tissue architecture [7].

During gastrulation, YAP activity contributes to the coordinated cell movements that establish the primary germ layers. Spatial regulation of Hippo signaling ensures that YAP is activated in regions undergoing dynamic remodeling, where reduced cell–cell contact, enhanced actomyosin contractility and integrin signaling favor its nuclear localization [123]. In these regions, YAP promotes transcriptional programs that facilitate processes essential for embryonic body plan formation, such as cell detachment and migration. Accordingly, studies in vertebrates show that gradients of tension and epithelial topology change the pattern of YAP activity required for proper germ-layer morphogenesis [124,125].

A particularly well-characterized example of type 1 EMT is neural crest development, where progenitor cells detach from the dorsal neural tube and migrate. In this context, YAP supports this cell behavior by coordinating cytoskeletal dynamics and responsiveness to environmental cues (integrins, RhoA/ROCK, and mechanosensitive channels such as Piezo1), thus ensuring proper cell distribution and differentiation [126,127]. Studies in zebrafish and chick indicate that YAP activity is required for neural crest progenitor proliferation/maintenance and for the expression of EMT effectors (SNAIL/SLUG, SOX9/10), whereas the loss of YAP/TAZ impairs delamination and migration [128,129,130].

YAP/TAZ also play a central role in the transduction of ECM signals during tissue morphogenesis. Embryonic tissues are mechanically heterogeneous, and YAP/TAZ activity is highly sensitive to variations in ECM stiffness, composition and organization. Through mechanotransductive pathways involving integrin-based adhesion, actomyosin contractility and apico-basal tension, YAP/TAZ convert ECM-derived mechanical information into transcriptional outputs that regulate cell shape, polarity, and fate [57]. This function is critical during morphogenetic events such as neural tube closure, organ budding (e.g., liver, pancreas, lung) and convergent extension, where coordinated changes in tissue stiffness and geometry guide collective directional cell movements and spatially restricted EMT behaviors [124]. In this way, YAP/TAZ act as sensor of the microenvironmental mechanics, ensuring that cells with EMT traits remain spatially confined for an appropriate development.

### 5.2. YAP/TAZ in Tissue Repair, Fibrosis and Regenerative EMT (Type 2 EMT)

In adult tissues EMT-related programs are reactivated in contexts of injury and repair, in an often partial and reversible form referred to as secondary EMT. Notably, during tissue regeneration, epithelial cells frequently undergo partial EMT states characterized by reduced adhesion, increased motility but also retention of epithelial features, thus enabling cells to migrate into damaged areas while preserving epithelial identity enough to allow later re-differentiation. Within this framework, YAP/TAZ act as key regulators [32].

The balanced plasticity of pEMT states is driven by transient Hippo pathway inhibition following injury-induced changes in tissue tension and architecture. In regenerative contexts such as liver and skin repair, YAP activation promotes controlled proliferation and migration, supporting wound closure without full loss of epithelial organization. Importantly, this transient activation is typically self-limiting, since the restoration of tissue integrity reactivates Hippo signaling and re-establishes YAP cytoplasmic sequestration [131,132].

Beyond driving single-cell EMT programs, YAP promotes collective migration, where cell clusters move coordinately while retaining partial cell–cell adhesion and epithelial architecture. YAP stabilizes a hybrid epithelial–mesenchymal state that preserves tissue cohesion while enabling motility and environmental sensing [133].

A central feature of YAP/TAZ function in secondary EMT is their extensive cooperation with the TGFβ/SMAD signaling axis [134,135]. TGFβ acts as a major inducer of repair-associated EMT and YAP enhances and stabilizes SMAD-dependent transcriptional programs. Nuclear YAP can form complexes with SMAD transcription factors, facilitating sustained expression of genes that can be involved in ECM production, cytoskeletal remodeling and cell migration [136,137,138]. This cooperation amplifies the intensity and duration of TGFβ responses, effectively integrating mechanical signals with cytokine-driven pathways. As a result, YAP acts as a molecular bridge between biochemical injury signals and mechanical remodeling of the tissue microenvironment, ensuring coordinated repair responses.

While transient YAP activation is essential for regeneration, sustained or dysregulated activation leads to disease. In chronic injury settings, persistent mechanical stress and continuous ECM stiffening maintain YAP in an active nuclear state, overriding normal Hippo-mediated feedback inhibition. This generally leads to prolonged expression of profibrotic genes, excessive ECM deposition and progressive tissue stiffening, which further reinforces YAP activation in a pathological feedback loop [139]. In fibroblasts, but also in epithelial cells undergoing EMT, this sustained signaling promotes differentiation into myofibroblasts, cells primarily responsible for scar tissue production and irreversible tissue remodeling characteristic of organ fibrosis [140].

The pathogenic role of TAZ in idiopathic pulmonary fibrosis (IPF) was investigated by Noguchi et al. [141] by using human lung histology and cell culture models. Functionally, TAZ governed lung fibroblast proliferation, migration, matrix and myofibroblast marker expression, while transcriptomic profiling identified TAZ as a key regulator of CTGF and type I collagen expression. Furthermore, Liu and Colleagues reported that matrix stiffness in the lung activates fibroblasts through the YAP/TAZ target PAI-1 that, in turn, reduces pericellular plasmin activity and this results in promoting cell–matrix adhesion and YAP/TAZ localization in the nucleus [142].

In vivo and in vitro models also demonstrated that YAP/TAZ drive the activation of hepatic stellate cells, responsible for massive deposition of ECM components in liver fibrosis [143,144,145]. Consistently, Hedgehog signaling was found involved in HSC transdifferentiation by its-mediated YAP activation [146]. Moreover, the TAZ-mediated Indian hedgehog (Ihh) gene induction, that is a key event in the transition from steatosis to non-alcoholic steatohepatitis (NASH), modulates profibrotic gene expression in HSCs, driving their transdifferentiation into ECM-producing myofibroblast [147]. Furthermore, in promoting renal fibrosis, YAP/TAZ mediate mTORC2-stimulated fibroblast activation [148]. YAP also exert a key role in maintaining skin homeostasis [149] and YAP/TAZ deletion interferes with keratinocyte differentiation and wound healing [150]. Coherently, YAP/TAZ show enhanced nuclear localization in wound-healing repair, while their downregulation affects timing of wound closure and reduces the expression of TGFβ1 and Smad2 [151]. Moreover, in skin, YAP1 is required to myofibroblasts differentiation [152] and the downregulation of YAP/TAZ signaling by PI3K/Akt1/GSK3β pathway prevents fibrosis in in vivo and in vitro models [153].

Overall, YAP/TAZ shift from regenerative regulators of secondary EMT to drivers of maladaptive repair, where the same plasticity mechanisms that enable wound healing become sources of chronic tissue dysfunction.

### 5.3. YAP/TAZ in Cancer-Associated EMT (Type 3 EMT)

In oncological contexts, EMT is frequently described as a pathological or tertiary transition, characterized by chronic activation, loss of homeostatic feedback control, and a profound integration into oncogenic signaling networks. Within this regulatory framework, YAP/TAZ function as pivotal drivers of phenotypic plasticity, integrating mechanical, inflammatory and oncogenic signals to maintain metastatic potential and pharmacological resistance. Throughout the metastatic cascade, YAP/TAZ are essential at multiple levels, including local tissue invasion, intravasation, survival within the systemic circulation and the eventual colonization of secondary sites [154]. Their activation facilitates the detachment of epithelial cells by suppressing cell–cell adhesion molecules, such as E-cadherin, while simultaneously stimulating the cytoskeletal reorganization necessary for active migration and invasion through the ECM. In primary tumors, YAP activity is often enriched in areas of significant mechanical stress where the inhibition of the Hippo pathway prevents the proteasomal degradation of YAP and triggers its nuclear accumulation. Consequently, malignant cells can transiently adopt EMT-like states that favor dissemination. Notably, analysis of circulating tumor cells (CTCs) in patients and animal models has revealed the concomitant expression of epithelial and mesenchymal markers, mainly in clusters of CTCs, where YAP contributes to the stabilization of a hybrid epithelial–mesenchymal state [133], supporting the role of YAP in tumor dissemination through the maintenance of partial EMT states in cancer cells [155,156].

In advanced disease stages, YAP further supports extravasation and the adaptation to metastatic niches by activating cell-autonomous survival circuits, upregulating anti-apoptotic factors and tuning the cellular response to local microenvironment signals [157].

A defining characteristic of YAP-mediated EMT in cancer is the acquisition of stem-like properties. The activation of YAP/TAZ initiates transcriptional networks linked to self-renewal, metabolic adaptation and long-term tumor maintenance [154]. Research from the Piccolo’s group has identified the YAP/TAZ axis as a fundamental rheostat of cell identity, proving that its activity is both necessary and sufficient for the dedifferentiation of somatic cells into tissue-resident progenitors and for endowing malignant cells with cancer stem cell (CSC) traits [20]. Rather than enforcing a static, terminal mesenchymal state, YAP/TAZ facilitate highly dynamic and reversible shifts between epithelial, mesenchymal and hybrid configurations [158]. Single-cell transcriptomic evidence supports a model where this hybrid plasticity serves as a primary determinant of intratumoral heterogeneity, treatment resistance and clinical severity [159]. Therefore, through synergistic binding with TEADs and collaboration with canonical EMT-TFs, YAP/TAZ maintain a permissive transcriptional environment that allows cancer cells to adapt dynamically to microenvironmental stressors and therapeutic interventions.

Simultaneously, YAP/TAZ promote immune evasion by regulating both intrinsic and extrinsic signaling pathways. Within neoplastic cells, active YAP signaling is associated with diminished antigen presentation through the suppression of MHC class I molecules [160] and the direct transcriptional induction of immunosuppressive ligands like PD-L1, which effectively impair T-cell recognition [161].

Extrinsically, YAP shapes the tumor microenvironment by triggering the release of specific chemokines, including CXCL5, which attract myeloid-derived suppressor cells (MDSCs) to block T-cell infiltration [162]. This is accompanied by the extensive activation of cancer-associated fibroblasts (CAFs) and subsequent changes in ECM deposition [163]. In turn, the increased tissue rigidity and physical tension stimulate mechanotransductive pathways that reinforce nuclear YAP signaling in both malignant and stromal cells [164]. Together, these mechanisms establish a self-perpetuating circuit between the tumor and its environment that favors immune escape, fibrotic ECM remodeling and persistent tumor growth.

## 6. Conclusions and Perspectives

### 6.1. The Emerging View: YAP/TAZ as a Central Hub of Cellular Plasticity in EMT

**Figure 1:**
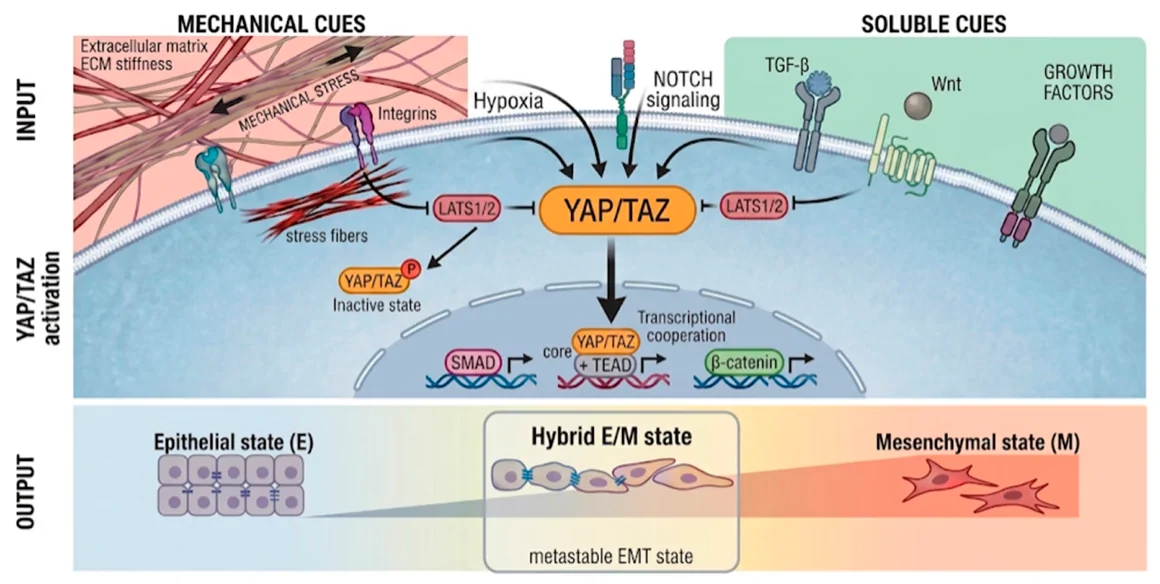
Schematic representation of YAP/TAZ as a central regulatory hub integrating extracellular mechanical and biochemical cues to maintain a reversible EMT continuum and sustain cellular plasticity. The microenvironmental cues converge on the Hippo pathway to control YAP/TAZ activity. When Hippo signaling is inactive, YAP/TAZ translocate to the nucleus, where they interact with TEAD and cooperate with transcriptional regulators such as SMADs and β-catenin to drive context-dependent gene expression. The resulting transcriptional programs promote epithelial–mesenchymal plasticity, supporting transitions between epithelial, hybrid E/M, and mesenchymal states. Additional Hippo-independent pathways and feedback mechanisms influencing YAP/TAZ dynamics are discussed in the text.

As explored throughout this review, YAP and TAZ do not merely function as passive downstream effectors of EMT-related signaling. Rather, they serve as dynamic regulators that actively direct cell-state transitions by coupling environmental information with the core transcriptional networks controlling EMT initiation, progression and maintenance. By functioning as a central regulatory hub, YAP/TAZ stand at the intersection of microenvironmental cues and transcriptional outputs, effectively directing cell fate and adaptive behavior (Figure 1).

The functional outcomes of YAP/TAZ signaling are highly context-dependent. During embryogenesis, YAP activity enables coordinated and reversible morphogenetic events guided by specific spatiotemporal signals. In contrast, during tissue repair and fibrosis, chronic mechanical stress and sustained ECM remodeling keep YAP/TAZ active, which often restricts phenotypic reversibility and favors pathological tissue remodeling. In cancer, the convergence of aberrant mechanical, inflammatory and immune-mediated cues results in persistent YAP activation, driving tumor invasion, metastatic dispersal, pharmacological resistance and the emergence of intratumoral heterogeneity.

### 6.2. YAP/TAZ and Hybrid Epithelial–Mesenchymal States

As previously outlined, EMT is increasingly recognized as a dynamic continuum of phenotypic states rather than a simple binary process. Within this model, hybrid epithelial/mesenchymal phenotypes represent metastable states that possess unique biological properties, including enhanced adaptability and plasticity compared to full epithelial or mesenchymal phenotypes.

In line with the mechanisms discussed here, YAP/TAZ appear to be key regulators of these hybrid E/M states. This is achieved through the integration of mechanical cues from actomyosin-mediated tension, Hippo-dependent and -independent signaling cascades and the EMT transcriptional program.

Variations in Hippo signaling intensity, together with dynamic changes in mechanical and biochemical inputs, can trigger the continuous nucleo-cytoplasmic shuttling of YAP/TAZ. This can conceivably result in gradual transcriptional outputs that favor the maintenance of hybrid states over discrete lineage programs. Such regulation likely allows for the coexistence of epithelial and mesenchymal traits, preserving the phenotypic flexibility required to adapt cells to changing microenvironmental conditions. According to this model, YAP/TAZ activity may represent a critical rheostat that determines whether cells remain in plastic intermediate states or progress toward stable epithelial or mesenchymal states.

Although YAP/TAZ signaling during EMT is predominantly sustained by positive feedback loops, YAP/TEAD have also been shown to induce negative regulators, including LATS2 and AMOTL2, which restrain YAP activity through homeostatic feedback mechanisms [165,166]. These intrinsic feedback circuits may contribute to preventing excessive YAP/TAZ activation and preserving the dynamic range of their signaling required for cellular plasticity. Nevertheless, whether such negative feedback mechanisms directly regulate EMT progression or specifically contribute to the maintenance of metastable hybrid E/M states remains to be established.

### 6.3. Clinical Implications of the EMT–YAP/TAZ Regulatory Circuit

The evidence presented here suggests that YAP/TAZ signaling not only facilitates EMT through transcriptional synergy with major pathways and transcription factors but also reinforces these states via positive feedback loops. These circuits involve matrix remodeling, integrin signaling and cytoskeletal reorganization, all increasing tissue rigidity and further sustaining YAP/TAZ nuclear localization and activity.

In pathological conditions like cancer and fibrosis, these mechanisms drive abnormal tissue remodeling and the persistence of activated mesenchymal-like populations, ultimately compromising organ architecture and function. Consequently, EMT and YAP/TAZ become locked in a self-perpetuating regulatory circuitry that stabilizes pathological cell states. In cancer, this stabilization promotes metastatic spread, stemness and resistance to diverse therapies. In fibrosis, the persistent activation of the EMT-YAP/TAZ axis maintains myofibroblast differentiation and excessive matrix deposition, leading to chronic tissue remodeling and organ dysfunction.

This feedback circuit presents significant therapeutic implications. Since EMT and YAP/TAZ are linked through reinforcing mechanical and inflammatory networks, targeting a single component can be insufficient. Accordingly, increasing evidence suggests that effective treatment may require a combinatorial approach that simultaneously addresses YAP/TAZ activity, EMT pathways and the mechanotransductive signals that sustain pathological cell plasticity [167].

Notably, preclinical models utilizing pharmacological inhibitors of the YAP/TAZ–TEAD complex, such as verteporfin [168], have shown success in attenuating EMT, stem-like traits and fibrotic progression [169,170], thus reinforcing the central role of YAP/TAZ in these processes and supporting their potential as therapeutic targets.

Collectively, the findings reviewed here support a model where YAP/TAZ act as the central molecular hub within the networks controlling cellular plasticity and EMT dynamic process. Future research aimed at clarifying the spatio-temporal and tissue-specific regulation of YAP/TAZ activity will be pivotal to understand how plastic cell states are established, maintained and reversed, potentially leading to more targeted therapeutic strategies for cancer, fibrosis and regenerative medicine.

## Data Availability

No new data were created or analyzed in this study. Data sharing is not applicable to this article.

## Abbreviations

The following abbreviations are used in this manuscript:

E/M	Epithelial/Mesenchymal
EMT	Epithelial-to Mesenchymal Transition
pEMT	Partial EMT
ECM	Extracellular Matrix
YAP	Yes-associated protein
TAZ	Transcriptional co-Activator with PDZ-binding motif
MST1/2	Mammalian Ste20-like kinases 1 and 2
LATS 1/2	Large tumor suppressor 1/2
EMT-TFs	EMT Transcription Factors
TGFβ	Transforming Growth Factor beta
PRRX1	Paired-related homeobox 1
CTCs	Circulating Tumor Cells
CSCs	Cancer Stem Cells
